# Economic Evaluations of the Health Impacts of Weather-Related Extreme Events: A Scoping Review

**DOI:** 10.3390/ijerph13111105

**Published:** 2016-11-08

**Authors:** Laetitia H. M. Schmitt, Hilary M. Graham, Piran C. L. White

**Affiliations:** 1 Centre for Health Economics, University of York, YO10 5DD York, UK; 2 Academic Unit of Health Economics, University of Leeds, LS2 9LJ Leeds, UK; 3 Health Sciences Department, University of York, YO10 5DD York, UK; hilary.graham@york.ac.uk; 4 Environment Department, University of York, Wentworth Way, YO10 5NG York, UK; piran.white@york.ac.uk

**Keywords:** climate change, heat waves, floods, hurricanes, economic evaluation, morbidity, mental health, mortality

## Abstract

The frequency and severity of extreme events is expected to increase under climate change. There is a need to understand the economic consequences of human exposure to these extreme events, to underpin decisions on risk reduction. We undertook a scoping review of economic evaluations of the adverse health effects from exposure to weather-related extreme events. We searched PubMed, Embase and Web of Science databases with no restrictions to the type of evaluations. Twenty studies were included, most of which were recently published. Most studies have been undertaken in the U.S. (nine studies) or Asia (seven studies), whereas we found no studies in Africa, Central and Latin America nor the Middle East. Extreme temperatures accounted for more than a third of the pool of studies (seven studies), closely followed by flooding (six studies). No economic study was found on drought. Whilst studies were heterogeneous in terms of objectives and methodology, they clearly indicate that extreme events will become a pressing public health issue with strong welfare and distributional implications. The current body of evidence, however, provides little information to support decisions on the allocation of scarce resources between risk reduction options. In particular, the review highlights a significant lack of research attention to the potential cost-effectiveness of interventions that exploit the capacity of natural ecosystems to reduce our exposure to, or ameliorate the consequences of, extreme events.

## 1. Introduction

An increase in extreme events, including their frequency, intensity and modifications to their spatial extent and timing, constitutes one of the many expected consequences of climate change [1]. Extreme weather has attracted increasing attention over the last 15 years in the aftermath of a series of highly-devastating events, including the European heat wave in 2003, hurricanes Katrina and Rita in the U.S. in 2005, a series of extensive floods in Japan (2007), Vietnam (2007), Pakistan (2010) and Bangkok (2011) and hurricane Sandy in 2012.

The economic consequences of weather-related extreme events are substantial, with estimates of annual costs ranging from $94 billion to over $130 billion globally [2]. In addition to causing considerable damages to assets and productive capital, the adverse effects of climatic extremes on human health figure prominently on the political agenda around addressing climate change risks [3]. This can be seen both as a response to public opinion, where adverse health effects have been identified as one of the main public concerns about climate change consequences [4] and an appreciation that health costs, in terms of individual welfare changes, but also healthcare resource use and labour productivity loss, are expected to make a substantial contribution to the overall economic impacts associated with a warmer climate [5,6].

Interestingly, however, as recently noted by the Intergovernmental Panel on Climate Change (IPCC) [7], studies of the economic impacts of climate change have essentially focused on impacts to infrastructure and tradable assets, as opposed to impacts on the health of humans and ecosystems, and when health effects have been considered (e.g., the PESETTA project in Europe [8]), they essentially pertain to a gradual increase in global mean temperatures, e.g., Bambrick et al. [9], Kovats et al. [10], Bosello et al. [11]. Not only do these studies not fully capture the burden of heat waves, due to non-linearity in risk at high temperatures and the cumulative effect of sustained heat load [12], but they ignore the health costs associated with other weather-related extreme events, such as droughts, floods and hurricanes. Furthermore, in a context where the effects of climate change are already being experienced, economic studies are needed not only to assess the size of the current and projected health costs of extreme events, but also, to evaluate the cost-effectiveness of risk reduction measures. Whilst the IPCC recently concluded that the evidence base on the economic efficiency of adaptation measures against climatic extremes is limited [7], the most recent review used to support this statement dates back to 2009 [13].

To our knowledge, beyond the economic evidence related to climate change in general, which has overwhelmingly focused on heat risk, there has been no review of studies that evaluate the economic costs of the adverse health effects associated with human exposure to extreme events. In addition, it is of particular interest to evaluate the latest state of the economic evidence base on risk reduction measures targeted at reducing population exposure to extreme events. We therefore conducted a scoping review of this important area of research. Scoping reviews are particularly suitable for conveying the extent and breadth of a given field of research that spans across methods and disciplines by using broad search terms and not applying quality filters [14,15,16]. Scoping reviews also involve an analytical interpretation of study findings [17], which helps underline the policy implications of current evidence, as well as directions for future research. In order to try to capture the diversity of the studies’ objectives and methodologies used, we did not apply any restrictions to the type of economic studies included in our scoping review, provided they incorporated some estimates of costs.

## 2. Methods

### 2.1. Search Terms and Databases

We searched the PubMed, Embase and Web of Science databases in December 2015, without date limitations, for papers in English that provide an economic evaluation of the health costs resulting from human exposure to weather-related extreme events. Search keywords were determined in agreement with all three authors after an initial broad search of the literature. They included a broad range of terms relevant to the varied nature of: (i) weather-related extreme events (search terms included: “extreme heat”, “extreme cold”, “hot temperature”, heat wave, heat, hot, flood, drought, smog, ozone, cyclonic storms, hurricane); and (ii) the health effects associated with them (search terms included: morbidity, mortality, death, hospitalization, illness, exposure, stress, post-traumatic). Since the focus of the review was specifically on economic studies, these two search fields were combined (AND) with the keyword “cost” in the title or abstract. Whilst other economic-relevant keywords, such as “economic”, or “burden”, or “losses”, were initially added to the search, they were dropped as they were not found to improve the accuracy of the search that was specifically looking for cost estimates, as opposed to a qualitative assessment of the magnitude of the economic burden of extreme events.

### 2.2. Inclusion/Exclusion Criteria and Identification of the Pool of Relevant Articles

Articles had to meet three inclusion criteria. First, they had to pertain to weather-related extreme events. This means that papers that solely produced projections of health burden and associated economic impacts under various scenarios of a warmer climate were excluded. Whilst air pollution is essentially the by-product of human activity, pollution peaks often result from the conjunction of high levels of pollutant emissions and meteorological conditions (e.g., high pressure systems); consequently, it was decided to include pollution peaks as weather-related extreme-events. Second, articles had to include health effects, i.e., not focus solely on non-health related impacts, such as damage to assets. Third, whilst no restriction was applied to the type of economic evaluation, articles had to provide economic information. To avoid over-restricting the pool of relevant studies, monetized health impacts did not represent an inclusion criterion. However, in order to be included, studies had to include cost estimates alongside health impacts, be it costs of adaptive measures, monetized damages to assets or productivity losses for instance.

**Figure 1:**
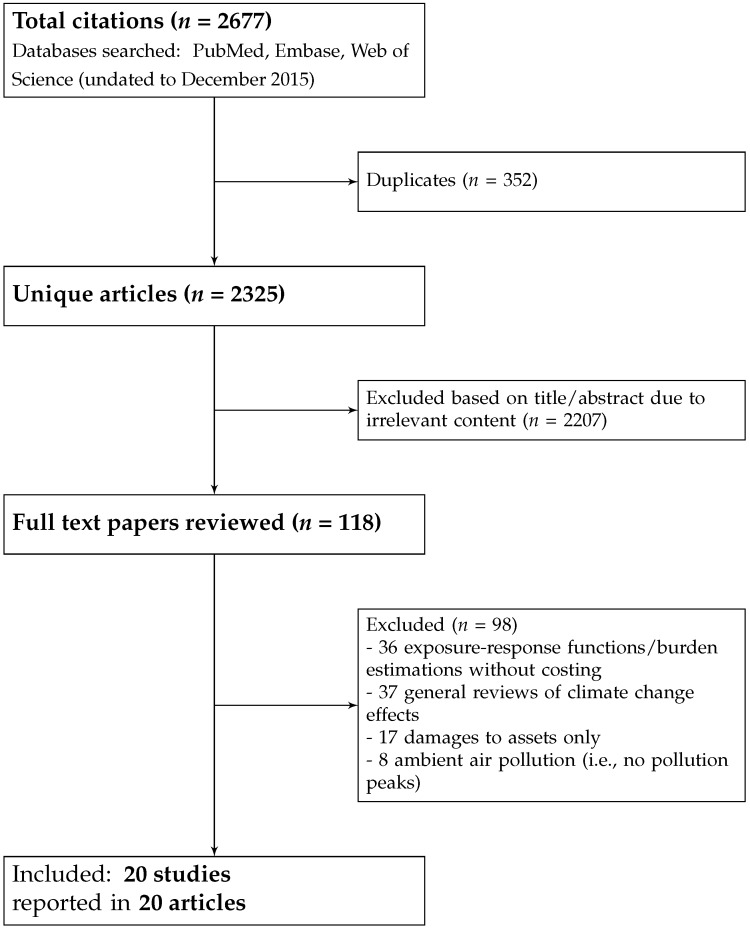
Flow chart of document selection.

The document selection process, underpinned by the three above-mentioned inclusion criteria, is summarized in Figure 1. After removing duplicates, the search led to the identification of 2325 distinct articles, 2207 of which were excluded by one reviewer (Laetitia Schmitt) based on title and/or abstract. These excluded articles were either on natural or mechanistic systems (*n* = 869) or their content was irrelevant to weather-related extreme events (*n* = 1338). Irrelevant articles pertained to five main categories: diseases not related to extreme events; medical therapies; burns; occupational heat exposure and ambient air pollution, since only peaks in exposure or alternatively, on pollution levels during heat waves were deemed relevant to the research question.

This led to the identification of a pool of 108 articles selected for full review, all of which could be retrieved. From this pool of articles, 98 articles were excluded after full review by Laetitia Schmitt and cross-validation with the other two authors (Piran White and Hilary Graham). More specifically, articles suggested for exclusion by Laetitia Schmitt were classified according to four main categories of reasons for exclusion from each of which Piran White and Hilary Graham independently took random samples of a minimum of five articles, in order to check for the validity of the exclusion decision. This cross-validation process was also performed for articles suggested for inclusion. For articles for which there was inconsistency between the three authors (*n* = 4), further discussions were held to reach consensus on inclusion or exclusion.

One category of articles excluded after full review was constituted of papers that only quantified the functional relationship between extreme event exposure and health effects and/or the burden of ill-health associated with extreme events, without encompassing any economic information (36 articles). A second category comprised general reviews, as well as studies of the burden of climate change that solely provided an evaluation of the effects under projections of changes in mean global temperatures, as opposed to effects associated with specific extreme events (37 articles in total; 2 of which [18,19] evaluated reduced GDP loss from reduced work capacity due to projected increases in thermal stress). A third category comprised articles that solely focused on damage to assets, property or crops and did not cover health effects (17 articles, mainly on flooding). A fourth category was constituted of articles pertaining to the health effects of indoor or ambient air pollution (8 articles excluded after full review, since most of these had been previously excluded based on abstract screening).

A total of 20 articles, corresponding to 20 distinct studies, were identified as relevant. Whilst no quality appraisal filters were applied, all but two articles were published in peer-reviewed journals. One paper [20] was a statistical bulletin and another [21] a publication from conference proceedings (see Table 1 in Section 2.3). Relevant articles came from all three databases searches, though a greater proportion came from Web of Science. Each relevant peer-reviewed article was published in a different journal and the main expertise areas they came from were: (i) disaster and preventive medicine; (ii) public health; (iii) environmental management.

### 2.3. Data Extraction

Each publication (i.e., study) was reviewed with respect to five key features: (i) general descriptive information, e.g., publication date, environmental hazard and geographical region of focus; (ii) type of research output, e.g., burden evaluation, economic appraisal, descriptive analysis; (iii) method used to measure and to monetize health effects; (iv) consideration of distributional effects; and (v) consideration of ecosystem services to help alleviate risk and damages. Data extraction was conducted by Laetitia Schmitt and checked by Piran White and Hilary Graham. The complete set of information extracted from each study can be found in Table 1.

## 3. Results

### 3.1. Publication Date, Environmental Hazard and Geographical Region of Focus

**Figure 2:**
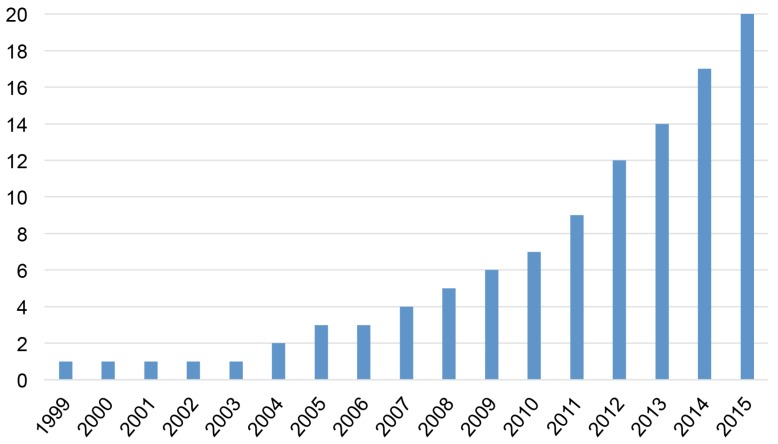
Cumulative number of studies providing an economic evaluation of the health impact of extreme events.

Despite the absence of date limitations in the search, the earliest relevant study was published in 1999, and two-thirds (13 out of 20) of the articles were published over the last five years (2011–2015). As Figure 2 indicates, the total number of relevant publications has increased seven-fold over the last decade. The implied growth rate is higher than the background increase in the global number of scientific publications (estimated at 5.6% per year over 1997–2006 for scientific publications in PubMed Medline [40]) and suggests that the economic evaluation of the health consequences of extreme weather events represents a very recent, but now rapidly-growing field of research.

An analysis of the distribution of the pool of identified studies according to their primary environmental hazard and geographical region of focus (Table 2) shows that some parts of the world are clearly under-represented. Most studies have been undertaken in the U.S. (nine studies out of 20) or Asia (seven studies out of 20), whereas no study has covered Central and Latin America, the Middle East nor Africa. This imbalance also explains that most studies (13 studies out of 20) pertained to high or middle-income countries; however, only one study was based in Europe (Spain).

Extreme temperatures (heat waves mainly) accounted for more than a third of the pool of studies (seven studies out of 20), closely followed by flooding (six studies out of 20). No economic study was found on drought. Whilst extreme temperatures have been considered in all but one region covered by the pool of identified articles (namely North America, Asia, Europe and Australia), flooding has been the main focus of studies in Asian countries and Turkey. All studies on hurricanes or tropical storms are from the U.S. whereas the two studies on pollution peaks are based on case studies in Asia (China and Malaysia, respectively).

### 3.2. Typology of Evaluation

**Figure 3:**
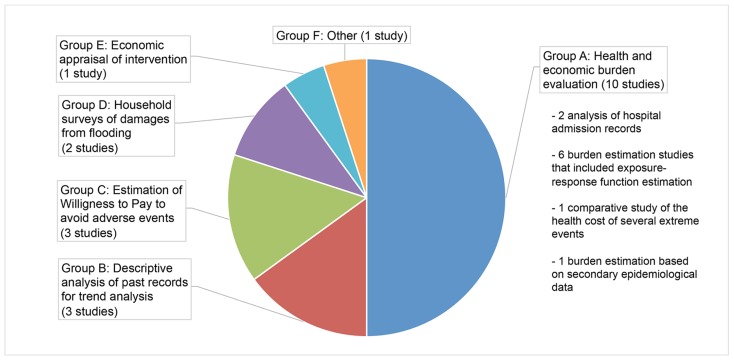
Typology of evaluation undertaken by the identified studies.

Studies were heterogeneous in terms of objectives and methodology and were classified into six categories—labelled A to E—as represented in Figure 3.

Half of the studies (*n* = 10) were classified as burden evaluation studies (Group A). They exhibited great variation in methods and could be further subdivided into four sub-groups (see Figure 3). Two studies [20,22] analysed hospital admission records for extreme temperature exposure, in order to provide an estimate of the healthcare cost burden associated with extreme heat and extreme cold. They also aimed to identify disparities in vulnerability to excessive temperature exposure between population subgroups stratified by age, gender and socio-economic status. Six studies performed an empirical estimation of the functional relationship between health effects and extreme event exposure using time-series data specific to their case studies, with a view toward estimating the associated health and economic burden. Three of these six studies focused on extreme heat [23,24,25], two on hurricanes [26,27] and one on smoke haze [29]. Among the three burden studies that focused on extreme heat as a health risk, two included projections of health and economic burden under IPCC climate scenarios [23,24]. The remaining two studies of Group A provided respectively: (i) a comparison of the burden associated with six types of climate-change related events (three of which were weather-related extreme events) that occurred in the U.S. over the period 2000–2009 [28]; and (ii) an estimation of the health and economic burden associated with the severe haze event of January 2013 in Beijing using secondary epidemiological data [30].

The three studies classified in Group B [31,32,33] provided descriptive statistics for trend analysis. They exploited records of extreme events fatalities and/or healthcare data and asset losses and aimed to assess whether trends in damages and loss of life were correlated with trends in the frequency and intensity of extreme events. Two of these studies [32,33] also investigated whether a change in meteorological conditions as a result of climate change could have influenced the frequency or intensity of extreme events and could explain the current upwards trend in fatalities and damages, whereas the third one [31] aimed at identifying seasonal trends in flood frequency, as well as the geographical areas that are the most prone to flooding in Turkey.

The three studies in Group C [21,34,35] estimated the willingness to pay (WTP) to avoid the adverse health impacts associated with extreme events using the contingent valuation method (see Section 3.5 for further explanations). Two of these WTP studies pertained to mental health effects and well-being reduction from flooding, whereas the third [35] focused on the excess risk of cardiovascular death from heat-stress and also estimated the functional relationship between these two outcomes using data for Taiwan. Two WTP studies were undertaken in high-income countries (Taiwan and Japan) and one in a low-income country (Vietnam).

Group D comprises two population-based surveys [36,37] that aimed to estimate the health and economic burden to households associated with flooding. Both surveys were undertaken in low-income countries (Pakistan and Bangladesh).

The study in Group E [38] is an economic appraisal of a risk reduction measure, namely a heat warning system in Philadelphia. The authors investigated the statistical relationship between excess deaths and heat wave warnings, in order to estimate the number of lives saved by the warning system, and compared the obtained benefits with the cost of running the system.

Finally, one study [39] — classified as “other” (Group F), draws from behavioural economics. This study tested for a potential relationship between (i) hurricane and tornadoes casualties and (ii) work routine and the embedded economic incentives in driving the adoption of risk protection measures at the individual level in the U.S.

### 3.3. Reliability of Study-Specific Results

Whilst scoping reviews do not apply quality filters to the body of the scientific evidence searched, because the studies were identified through a search of databases of peer-reviewed papers, a de facto quality filter was imposed.

The significance and reliability of results from the identified studies will greatly depend on the specific strengths and limitations of the method and datasets used. For instance, the reliability of burden estimates (Group A) from studies that relied on an empirical estimation of the functional relationship between exposure and effect using time series data specific to their case studies is expected to be higher than estimates based on secondary epidemiological evidence (all else being equal), since extreme events are very context specific and existing epidemiological data may not be easily transferable to different contexts and populations.

The significance of descriptive studies results (Group B), which only exploited correlation between selected variables of interest, will greatly depend on the temporal and spatial scale of the data and on the accuracy of the records (e.g., missing values). The three descriptive studies of Group B used data for extensive time-periods (≥25 years), in order to evaluate time trends in impacts at the country (Turkey, U.S.) or regional level (eastern Black Sea basin).

The main limitation of WTP studies based on stated preference methods (Group C) is that they rely on hypothetical scenarios that may lead to several biases. Nevertheless, the presently included WTP studies implemented innovative approaches in an effort to address the validity issues deemed most relevant to their specific case study. Matsushima et al. [21] valued WTP to avoid mental damages from flooding using an option value approach, in order to address potential strategic bias that would lead to an over-valuation of WTP. The WTP study of Navrud et al. [34] in Vietnam estimated the willingness to contribute in labour, in order to circumvent the fact that most individuals would not be able to afford any financial payment.

Population-based surveys results (Group D) may suffer from recall and/or strategic bias, where interviewees may exaggerate the severity of their losses if they believe this might help them to obtain further assistance.

The only economic appraisal study (Group E; [38]) identified is a very simple comparison of costs and benefit cumulated over three years without applying any discounting. It is worth noting that in this study, the association between heat wave warning and excess mortality was not statistically significant at the 5% level, but given the low cost of running the heat wave system, the large uncertainty around the expected health benefit was deemed acceptable.

Finally, the statistical study constituting Group F [39] provides an innovative analysis of U.S. records of hurricanes and tornadoes and their associated losses and casualties, but may suffer from a lack of household-based variables to provide a deeper understanding of the drivers of evacuation and sheltering behaviours.

### 3.4. Health Outcomes and Metrics Used

The health impacts of extreme events can be categorised into direct effects, including injuries, deaths and mental health difficulties and indirect effects that result from the primary damage [2]. This includes, for instance, waterborne or foodborne diseases as a result of, respectively, flooding or heat waves.

Most studies focused on direct impacts. The four main types of health metrics used were: (i) deaths; (ii) hospital admissions and outpatients visits; (iii) cases of acute morbidity or injuries; and (iv) depressive disorders or reduction in well-being. Death was used to measure the health burden associated with all four categories of extreme events represented by the selected studies, i.e., hurricanes, flooding, heat waves and air pollution peaks. By contrast, mental health problems were solely considered with regards to flooding (two studies; [21,34]) and, to a lesser extent, hurricanes (one study; [26]). In addition, hospital admissions were extensively used to measure the impact of extreme temperatures and peaks in air pollution.

Only one study [27] specifically evaluated indirect effects by investigating the effect of a heavily-damaged health infrastructure in the aftermath of hurricane Katrina on the long-term health outcomes of diabetic patients in Louisiana. This was also the only study that used a summary measure of population health (quality-adjusted life expectancy impacts) as a health outcome, whereas all of the other studies reported various health impacts separately, i.e., un-aggregated.

### 3.5. Approach to Monetization of Health Impacts

The total welfare effect associated with adverse health end-points typically encompasses three elements: (i) healthcare resource use; (ii) productivity loss; and (iii) dis-utility from suffering or life-shortening, where the latter component commonly drives the welfare loss associated with premature death in older people [12]. The money value of dis-utility associated with an adverse health outcome is typically informed by wealth-health trade-offs that individuals either reveal in surrogate markets (such as risk premiums in the job market) or state in hypothetical markets, as is done via contingent valuation or multiple choice experiments. The result is referred to as the willingness to pay (WTP) to avert outcomes or, when considering mortality risk, the value of a statistical life that is derived from individuals’ aggregated WTP for a small change in survival probabilities [41].

Out of the 10 studies that used death to quantify health impacts, only four applied a monetary value to this outcome by multiplying it with a value of statistical life. This is not surprising given that monetizing death is less useful for descriptive studies investigating trends in effects or for studies reporting results from population-based surveys (which represent five studies in our review).

With the exception of the studies that specifically aimed to estimate the WTP for morbidity risk reduction [21,34], the monetization of morbidity impacts was found to depend on the perspective of analysis chosen, though the latter was often not clearly stated. When burden analysis was undertaken from the perspective of the health care system [20,22,24,25,27], morbidity impacts were monetized using healthcare costs. In this case, mortality effects, if evaluated, where not monetized. Alternatively, when a broader societal perspective of analysis was chosen, [23,28,29,30], the cost of morbidity included lost productivity alongside healthcare costs. Whilst such an approach is commonplace in cost-of illness studies, it is worth underlining that it implicitly assumes that the loss of quality of life (or the dis-utility) from morbidity is nil and thus places a lower-bound on the total welfare effect of morbidity.

### 3.6. Distributional Assessment

Nine studies, [20,22,23,24,26,27,34,36,39], paid particular attention to the distribution of impacts based on demographic and socio-economic factors, such as age, gender, race/ethnicity and income and/or focused on specific population subgroups expected to be most vulnerable to extreme events. Vulnerable population subgroups considered, which may overlap, were: (i) older individuals (aged 65 and above) and disabled people (i.e., Medicare population in the U.S.); (ii) single parents; (iii) patients with a chronic condition (diabetes); and (iv) low-income communities. The large majority of the studies that examined the distribution of effects within population subgroups were undertaken in the U.S. (six out of nine studies); two were carried out in Asia [34,36] (focusing on low income communities only); and one in Australia [24] (focusing on age-related differential susceptibility to heat effect).

### 3.7. Consideration of Ecosystem Services to Help Alleviate Risk and Damages

There is a substantial literature on the protective benefits of natural ecosystems, such as coastal wetlands, riparian forests and reefs, against the devastating effects of flooding and hurricanes [42,43,44]. In addition, urban trees have been shown to help alleviate air pollution in cities by absorbing atmospheric pollutants [45] and reduce city heat island effects by lowering temperatures [46]. Despite this evidence and whilst a number of the included studies recommended the adoption of risk reduction measures, only one study on flooding [32] briefly suggested investing in natural ecosystems (watershed management) as part of a portfolio of adaptive measures.

## 4. Discussion

### 4.1. Key Findings

#### 4.1.1. Health Impacts Represent an Substantial Economic Burden That Is Likely to Rise Steeply

All studies indicate that the economic cost associated with the adverse impacts of extreme events on human health is substantial. Whilst it is not the aim of this review to report all quantitative estimates, key economic findings reported by each study can be found in Table 1.

The comparative study by Knowlton et al. [28] of the health costs associated with a range of six climate change-related events that happened in the U.S. between 2000 and 2009, ranked heat waves as the most costly weather-related extreme event ($5.4 billion (bn)) for California’s two-week long heat wave in 2006). Heat waves indeed claim substantial death tolls and a large number of emergency department visits due to a large exposed population. The interpretation of this ranking, however, requires caution. First, it was based on a selection of a single case study for each type of extreme event, which may explain why, in contrast to Knowlton et al. [28], other authors have ranked flooding as the weather-related extreme event with the greatest health impacts [47]. Second, only direct health impacts were accounted for in this study. Accounting for indirect health effects over a longer time horizon, such as reduced healthcare provision following damage to infrastructures (see Section 4.1.2), may provide a slightly different picture. Third, this ranking may be different in low-income countries, where the number of deaths from flooding and hurricanes is greater than in high-income countries.

More importantly, the two studies that included burden projections under various climate scenarios [23,24] show that, whilst unavoidably highly uncertain, the health and economic burden of extreme events is set to rise steeply under projected increases in mean temperatures globally. For instance, Lin et al. [23] estimated that the annual healthcare costs from heat-related respiratory admissions in New York city currently amounts to $0.64 million (m) but is projected to surge to $5.5–7.5 m p.a. in 2046–2065 and to $26–76 m p.a. in 2080–2099.

#### 4.1.2. Health Consequences May Be Incurred Long after Event Occurrence

Whilst extreme events are typically brief, their consequences for public health may last over long time horizons. For instance, Fonseca et al. [27] estimated that the disruptive impact of hurricane Katrina on the healthcare management of patients with diabetes would lead to a $504 m healthcare bill over the patients’ remaining lifetimes (discounting at 3% p.a.). This finding was based on the expected increase in incidence of co-morbidities and health complications in patients with diabetes, following medication deprivation during the shut-down period of local medical facilities. Furthermore, Zahran et al. [26] found that, in the aftermath of hurricanes Katrina and Rita in 2005, not only did single mothers experience a very high mental strain but, unlike the general population, they did not return to their pre-disaster mental health levels more than a year after the event.

Although the indirect long-term consequences of extreme events are potentially large and, in some cases, expected to substantially contribute to the total economic impact of these events, they appear to be rarely examined (two studies out of the pool of relevant studies). The reason is that, since these impacts are temporally separated from event onset, it is much more resource-intensive to capture them, requiring for instance follow-up surveys or modelling expertise to extrapolate health consequences later in life.

#### 4.1.3. An Increased Vulnerability to Events Exacerbated by Human Factors

The analyses of Yüksek et al. [32] and Kunkel et al. [33] suggest that the upwards trend in adverse health impacts and asset damages from extreme events witnessed over the last few decades essentially results from increased vulnerability, as opposed to changes in atmospheric conditions. Whilst the factors that exacerbate vulnerability to extreme events will vary geographically, they typically include a growing population in coastal areas, land use modification (deforestation) due to rapid urbanization and an ageing population in developed countries.

Economic factors were also found to play a major role in shaping people’s attitudes towards the risk of extreme events. For instance, Yüksek et al. [32] suggest that the rise in land and property prices in the eastern Black Sea basin in Turkey has pushed people to settle on riverbanks, despite known flood risk. In addition, the results of Zahran et al. [39] support the hypothesis that the cost of adopting protection measures, such as income loss, influences individuals’ evacuation behaviour when informed of hurricane risk.

#### 4.1.4. Disparities in Vulnerability between Population Subgroups Is Expected to Exacerbate Health Inequalities between Income Groups

A number of population subgroups, such as older people, single mothers, patients with a chronic condition and socio-economically disadvantaged communities, was found to bear a disproportionate health burden associated with extreme events. Whilst the review does not represent the overall body of evidence on vulnerability factors, it is worth noting that its findings are in line with the literature on the distributional effects of extreme events, heat-stress in particular [12,48].

All four studies that investigated the interactional effect of age and extreme temperatures found an increased vulnerability to heat-stress associated with age [20,22,23,24], with a significantly higher rate of hospital admissions in older age groups. By contrast, evidence of gender-related susceptibility to extreme temperatures is more mixed. Males were found to be at greater risk of hospitalization for both hypo- and hyper-thermia [20,22], whereas females were found to bear a disproportionate burden of respiratory admissions due to excess heat [23].

Low income was also found to be a factor associated with vulnerability. Merrill et al. [20] reported that hospitalization rates in U.S. hospitals due to extreme temperatures were 2 to 2.5 times higher in the poorest communities than in the wealthiest ones. This was corroborated by the findings from Lin et al. [23] who found an significant increase in the risk of respiratory admission under extreme heat among neighbourhoods with a high proportion of individuals with a low income. Importantly, the effect of income on vulnerability is not limited to extreme temperatures. Zahran et al. [39] for instance, found that, at the county level, the casualty risk from tornadoes decreased by 6% to 8% for every additional $1000 per capita income.

People on low income are expected not only to be more vulnerable to direct short-term effects but also to suffer a disproportionate burden of indirect consequences of events in the long run. In the U.S., Zahran et al. [26] showed that after hurricanes Katrina and Rita struck the U.S. Gulf coast in 2005, single mothers experienced a significantly higher number of days of poor mental health than the general population. When translated into productivity loss due to absenteeism, this additional mental strain represented an expected private income loss of $4,200 per single mother, as opposed to $817 for the average person. Fonseca et al. [27] demonstrated that the health of patients with diabetes who were treated in a state-funded system were more severely affected by hurricane Katrina than the health of patients treated in private or veterans-only health care systems, reflecting longer healthcare service disruption. As a result, the long-term healthcare cost impact of the hurricane is expected to be significantly higher for patients treated in state-run systems than in private systems. Given the tighter resources constraints that state-systems face, the hurricane is expected to have long-lasting implications for SES-related health inequality.

In developing countries, poorer households and households with livelihoods dependent on the exploitation of natural resources are expected to be more vulnerable to extreme events, such as floods, as the associated damage makes up a significantly larger portion of their annual income [34]. With regards to the distribution of health impacts, based on the 2011 Greater Bangkok floods case study, Nabangchang et al. [36] did not find a difference in flood-related injuries and illnesses between income groups. However, they found that evacuation rates did vary by income, with 77% of non-poor households having some members evacuate compared to 65% of poor households, which may suggest a greater adaptive capacity in non-poor households. In addition, whilst the authors did not provide the distribution of health-related costs (i.e., medicines, doctors’ visits and foregone income of patients and caretakers) by income strata, they reported a great variation in health costs incurred by households. Such a skewed distribution of healthcare costs impacts may further exacerbate wealth inequalities within the community.

### 4.2. Relevance of Currently-Identified Evidence for Policy-Making

It is worth restating that the review exclusively focused on economic evaluations of adverse health effects resulting from human exposure to extreme events. It therefore did not aim to review the overall evidence on the functional relationships between exposure to the various types of extreme events and health effects. Similarly, owing to its focus on extreme events, the review did not encompass the growing number of studies that provide projections of the health and economic burden associated with a future global rise in temperatures. It is nevertheless of interest to note that this excluded literature, namely exposure-response functions or health burden projections under climate change scenarios, has so far overwhelmingly focused on heat stress as a risk factor [49].

Whilst the databases searched are the ones widely used in the health and environment fields, by holding information on published peer-reviewed research, they are likely to be biased toward studies in high-income countries, thus leading to an under-representation of the body of “grey” research in low-income countries. In contrast, we do not consider that the exclusion of non-English language papers will have introduced a publication bias into our review. While we did not collect information on the number of non-English papers, a recent systematic review of extreme water-related weather events and waterborne disease that searched standard databases and included English and non-English language papers found that only 5% of the papers were not in English, and this small group was in other European languages [50].

Collectively, the studies in our review, which cover a diverse set of case studies, represent a valuable body of evidence on the size of the economic burden associated with the adverse health effects caused by exposure to weather-related extreme events. Alongside the literature on the health effects of climate change, the identified studies also provide policy-makers with an indication of how much this burden may grow by the end of the century, especially with regards to extreme temperatures.

In line with the wider literature on natural disasters [2], our review indicates that the recent upwards trend in adverse health impacts and asset damages from extreme events can be related in broad terms to demographic and lifestyle factors (e.g., population ageing, growing population in coastal areas) and approaches to natural resource management (e.g., land use modification), as opposed to climate change-related physical forcing. As changes in meteorological conditions become more pronounced under climate change, lifestyle and resource management factors are therefore expected to further exacerbate our vulnerability to extreme events. This review suggests that the modification of these factors should be at the centre of strategies aiming at reducing human exposure to extreme events.

Two studies identified in this review [26,27] have highlighted that extreme events can have long-term repercussions, especially on the health of vulnerable population subgroups. This is of particular interest as it can help identify the appropriate time-horizon for the economic evaluations of adaptive interventions, such as the implementation of protective measures.

Whilst most of the research on the health and associated economic effects of extreme events has focused on physiological impacts (e.g., deaths, hospitalizations), the recent attempts at valuing well-being and mental health effects will help provide a more comprehensive picture of the range of economic impacts. Efforts to estimate individuals’ welfare loss from mental health effects associated with exposure to climatic extremes should be particularly encouraged in light of growing evidence that these effects may be substantial [7] and the fact that, as mentioned in Section 3.5, valuing morbidity impacts solely based on healthcare resource use and/or productivity loss implicitly assumes that the quality of life loss from morbidity is nil.

Finally, the studies contribute to the larger body of evidence on the factors driving the distribution of adverse impacts and to the identification of vulnerable population subgroups stratified by demographics (e.g., age gender, socio-economic status) and health condition. These findings are key to the design and implementation of adaptation measures and, more generally, to inform the development of “healthy public policy”, which calls for an explicit consideration of health and equity matters in all policy areas [51].

### 4.3. Future Research Directions

The recent increasing research focus on the economic implications of the health impacts from extreme events (see Figure 2) coincides with an increase in the occurrence of weather-related disasters [52,53], linked to growing densities of population and assets in at-risk areas. Although this demonstrates a growing awareness of the need to document the economic burden associated with human exposure to extreme events, the body of evidence remains very thin and has so far largely focused on Asia and North America.

In particular, despite wide search terms and a long review period (undated to December 2015), we found no relevant study in Africa, Latin and Central America and the Middle East and only one study in Europe. This is an important gap, given that some of these areas are likely to experience severe impacts from climate change-related extreme events [7,54,55] and would highly benefit from a greater appreciation of the associated economic costs and their distributional implications. Whilst we acknowledge that the focus on published peer-reviewed research inherent to the databases searched may have led to an under-representation of research in low-income countries, this is unlikely to explain the paucity of studies in Europe. In addition, the lack of relevant economic studies in Africa is consistent with IPCC’s recent conclusion that information on the observed frequency of extreme events in Africa still remains limited [7].

Although decision-makers require evidence on the economic consequences of the public health burden associated with extreme events, the greatest evidence gap is in economic evaluations of possible interventions to reduce this burden, in order to allocate constrained resources towards the most cost-effective ones. One striking finding of this review is that despite the WHO’s 2009 call for further research on interventions to control climate-sensitive health risks and the fact that the large majority of the pool of included studies were published in the last five years, only one out of the 20 economic studies identified was an economic appraisal of a risk reduction measure. This economic evaluation pertained to a heat warning system [38] and was a rather crude comparison of costs and benefits cumulated over a three-year period.

This finding is corroborated by results from a recent systematic review by Bouzid et al. [56], which highlighted a paucity of evidence on the effectiveness of public health interventions aimed at reducing the health risks related to a changing climate. The authors identified droughts and floods among the climate risks for which there was no review of evidence on the effectiveness of potential interventions to reduce the associated public health burden. Although the authors found two reviews of evidence related to the management of heat stress, one did not pertain to heat waves, while the other included studies of very disparate quality. The expected increase in the intensity and the frequency of weather-related extreme events [1] adds urgency to the need to fill the evidence gap pertaining to the economic appraisal of adaptive measures.

Our review also indicates that very little research attention has been given to the public health dimensions of natural resource management and, more specifically, to the exploitation of the capacity of natural ecosystems to reduce human exposure to extreme events. This is a highly significant gap in the evidence base, in light of evidence that poor land management has been identified as a key factor exacerbating our vulnerability to extreme events (see Section 4.1.3). There is an urgent need to consider the restoration of various natural ecosystems, while taking into account non-linearity and scale-dependence in ecosystem services provision [43,57], within the scope of potentially cost-effective risk reduction options.

Finally, the building of a body of economic evidence to support resource allocation between competing strategies to adapt against a given type of extreme event would require a common methodological framework with regards to the range and duration of health impacts included and the approach to monetization. The choice of the range and duration of impacts are related, whereby indirect impacts typically occur in the long run. The identification of the appropriate analysis time horizon should therefore assess whether indirect effects, such as treatment disruption following damages to infrastructure following a hurricane or delay in care provision due to peaks in healthcare demand during a heat-wave, are likely to be influential to the analysis. Finally, the evaluation of competitive interventions should ideally be undertaken from the same perspective, e.g., healthcare system or society, as the chosen perspective of analysis will drive the approach to monetizing health effects (see Section 3.5).

## 5. Conclusions

Our scoping review has shown that, although the last five years have seen increasing research interest in the economic consequences of the health effects associated with human exposure to extreme events, the evidence base remains thin with limited geographical coverage. We found no studies in Africa, Latin and Central America and the Middle East, a critical gap given the projected distribution of climate-related extreme events for the future.

The economic studies identified were heterogeneous in terms of objectives and methodology and provided a mix of descriptive analyses, WTP estimation for health risk reduction, burden estimation and economic appraisal. However, when considered altogether, they clearly indicate that extreme events will increasingly become a pressing public health issue with strong welfare and distributional implications. Health impacts and associated consequences on healthcare budgets, productivity and individuals’ well-being may be incurred long after event occurrence and are expected to exacerbate health inequalities between income subgroups.

Whilst the evidence base identified by our review underlines the importance of addressing the health impacts of extreme events, it provides policy-makers with little economic information on which to base decisions about the allocation of scarce resources between potential risk reduction options. In particular, our review highlighted a significant lack of research attention to the potential cost-effectiveness of interventions that exploits the capacity of natural ecosystems to reduce our exposure to extreme events, despite evidence that poor land management contributes to exacerbating our vulnerability to them.

## Figures and Tables

**Table 1:** Information extracted from the pool of relevant studies.

Authors (Date)	Event Type	Country	Time-Period/Case Study	Pop	Study Objective & Method	Typology	Health Metrics	Health Impacts Monetization	Ecosystem Services Mentioned?	Distributional Analysis	Key Results
[20] (2008)	Extreme temperatures	U.S.	2005	All	Descriptive analysis of statistics on hospital stays resulting from excessive heat or cold exposure due to extreme weather conditions.	Burden evaluation (Group A)	Hospitalization admissions	Healthcare costs	No	Yes	Excessive temperatures costed U.S. hospitals $120 m in 2005. The average cost of a heat-related stay amounted to $6200 vs. $12,500 for cold-related stay. Patients admitted were older than the average hospital patient (6 to 7.6/100,000 people aged ≥65 years old). Hospitalizations were found to be about 2 to 2.5 times more common in the poorest communities than in the wealthiest ones and slightly more common in rural regions.
[21] (2007)	Flooding	Japan	2004 Toyooka flood	All	Estimation of willingness to pay (WTP) (contingent valuation) to avoid of mental damage caused by flood disaster (indirect approach using option value).	WTP study (Group C)	Mental damage	WTP estimated in the study	No	No	Individuals expressed a significant WTP to avoid mental damage: Mean WTP 44,769 yen.
[22] (2012)	Extreme temperatures	U.S.	2004–2005	U.S. Medicare pop. (≥65 years old or disabled)	Descriptive study to assess the health care burden of hypo- and hyper-thermia due to extreme weather conditions.	Burden evaluation (Group A)	Inpatient and outpatient visits	Healthcare costs	No	Yes	Hyperthermia-related visits were more frequent than hypothermia but less costly ($36 m vs. $98 m for hypothermia in 2004–2005). Black and Native Americans had a significantly higher relative risk of healthcare visits than their white counterparts.
[23] (2012)	Extreme temperatures	U.S.	1991–2004 for current burden; 2046–2065 and 2080–2099 for projections	All	Estimation of current and projected heat-related public health burden in New York State under a range of three IPCC climate scenarios.	Burden estimation (Group A)	Respiratory admissions	Healthcare costs and productivity loss due to days hospitalized. Adjustment for inflation and 3% discounting used. Costs normalised to $2004.	No	Yes	Hospital costs associated with heat-related respiratory admissions in NYS are currently estimated at $0.64 m p.a. and projected to increase to $5.5–7.5 m in 2046–2065 and to $26–76 m in 2080–2099. The public health burden is projected to be greater among females and in low-income groups.
[24] (2015)	Extreme temperatures	Australia	2000–2010 for current burden; years 2030 and 2060 for projections	All	Estimation of the current and projected burden of heat-related emergency department visits in Brisbane under a range of two IPCC climate scenarios.	Burden estimation (Group A)	Emergency department (ED) visits	Healthcare costs (normalised to AU$ 2013)	No	Yes	Higher relative risks of ED visits for adults aged 65+ than for their younger counterparts (RR for all ED visits = 1.09 vs. 1.06) on hot days (>35 degrees). ED visits are projected to increase considerably on hot days in the future under population growth and climate change scenarios. The excess number of visits by older patients is estimated to grow twice as much as the younger group. The excess demand is estimated to add an extra cost of around AU$78,000–260,000 in 2030 and AU$215,000–1,985,000 in 2060 (2013 prices).
[25] (2015)	Extreme temperatures	Spain	2002–2006	All	Estimation of: (i) the impact of excessive heat on mortality; (ii) the temperature threshold to mortality increase; (iii) the hospital cost of heat-attributable deaths.	Burden estimation (Group A)	Deaths	Healthcare costs	No	No	A statistically significant increase in mortality was observed when daily max temperature reached 38 ${}^{\circ }$C degrees. Over 2002–2006, excessive heat was found to be responsible for 107 (95% CI: 42–173) premature deaths, associated with a healthcare cost of €426,000 (€167,000–689,000).
[26] (2011)	Hurricanes	U.S.	Hurricanes Katrina and Rita in 2005	Single mothers	Estimation of the impact of exposure to hurricanes on the mental health resilience of single mothers versus the general population and computation of the related economic cost from lost productivity.	Burden estimation (Group A)	Days of poor mental health (reported in the last 30 days after event)	Direct private costs from absenteeism due to mental health disturbance	No	Yes	Following exposure to hurricanes Katrina and Rita, days of poor mental health was found to increase by 72% in single mothers vs. 18% in the total population. As a result, single mothers were expected to be absent from work 18.4 more days (vs. 3.6 more days of absence for the average person), leading to an income loss of $4.200/person (vs. $817 for the average person), thus exacerbating their economic vulnerability. Differential effects were found to persist one year after the events.
[27] (2009)	Hurricanes	U.S.	Hurricane Katrina in 2005	Patients with diabetes	Observational before/after study of the impact of Katrina on healthcare management of patients with diabetes in 3 different healthcare systems and projected health and healthcare costs consequences of treatment disruption over patients remaining lifetime.	Burden estimation (Group A)	Life expectancy (LE); quality-adjusted life expectancy (QALE)	Health care costs from treatment disruption over patients’ remaining lifetime	No	Yes	Treatment disruption in patients with diabetes following Katrina was projected to result in substantial health care costs ($504 m for the affected pop.) due to co-morbidities/disease complications in the long run. The impact reflects the high prevalence of the disease (about 9% of U.S. pop.) and the large size of the population affected. The disaster exacerbated inequalities in access to healthcare and resulting health disparities between socio-economic subgroups.
[28] (2011)	Multiple events	U.S.	2000–2009	All	Estimation and comparison of the health costs associated with 6 climate change related-events : (i) California heatwave 2006; (ii) ozone air pollution (for daily levels above national standards; impacts computed for the years 2000–2002); (iii) Florida hurricane season 2004 (4 hurricanes in one month); (iv) West Nile virus outbreak (vector-borne disease) in Louisiana; (v) red river flooding in North Dakota in 2009; (vi) Southern California Wildfires in 2003.	Burden study (Group A)	Deaths, hospitalizations, emergency department visits and outpatient healthcare use	VSL for mortality ($7.8 m in $2008); healthcare costs and loss-work productivity for morbid endpoints	No	No	Events associated with the greatest number of premature deaths were associated with the highest costs. The costliest weather-related extreme event in terms of health impacts was California’s 2006 heat wave ($5.4 bn), followed by Florida hurricane season ($1.4 bn), California wildfires ($600 m), West Nile infectious disease outbreak ($207 m) and red river flooding ($20 m). When normalised to 1000 people, the cost of river flooding was, however, nearly as high as the cost of heatwave ($150 k/1000 person).
[29] (2014)	Pollution peak	Malaysia	2004–2009	All	Estimation of the change in hospital admissions for a change in pollution concentrations (dose-response function) and evaluation of the associated economic burden.	Burden estimation (Group A)	Respiratory and cardiovascular hospital admissions	Healthcare costs and productivity loss	No	No	On average, over 2004–2008 for Kuala Lumpur and some areas in Selangor state (equivalent to 25% of the Malaysian population), smoke haze occurrences were found to be associated with an increase in inpatients visits by 2.4/10,000 people per year, representing a 31% increase from normal days. The associated economic loss amounted to $91,000 per year. Under no change in haze recurrence, over 20 years, this would represent a cost of $1.7 m, discounting at 5% p.a.
[30] (2015)	Pollution peak	China	Severe haze event in January 2013	All	Modelling of $PM_{2.5}$ concentrations during the haze episode and estimation of the associated acute mortality and morbidity impacts and associated health care costs.	Burden estimation (Group A)	Deaths, cases of acute bronchitis and asthma, hospital admissions	VSL for mortality ($274 k); WTP or healthcare costs and productivity loss for morbid endpoints	No	No	The total economic cost of the haze-related health impacts was estimated, under conservative assumptions, at $253 m, i.e., about 0.8% of the annual GDP of Beijing.
[31] (2005)	Flooding	Turkey	1970–1996 (624 floods recorded)	All	Descriptive analysis of seasonal and regional trends in the mortality and economic impacts of flooding based on registered flood reports.	Descriptive analysis (Group B)	Deaths	N.A. (see Section 2.2 and Section 3.5)	No	No	Seasonal and regional trends in terms of human deaths and economic impacts were determined. Most floods and deaths happened in the summer season. Most of the floods and deaths occurred in the Black Sea region.
[32] (2013)	Flooding	Turkey	Large floods between 1955–2005 in the eastern Black Sea basin (EBSB)	All	Descriptive analysis of flood occurrence and meteorological conditions and identification of trends in damages and human lives loss.	Descriptive analysis (Group B)	Deaths	N.A. (see Section 2.2 and Section 3.5)	Yes	No	Between 1995–2005, 51 floods occurred in EBSB causing 258 deaths and $500 m of damages to assets. Most floods occurred during summer months when snow melt is combined with heavy rainfall in mountainous valleys. Despite the absence of an increasing trend in extreme rain values and flood frequency, an upward trend in terms of both death and damages was found. The latter was attributed to human factors, such as illegal land use, urbanization in flood-prone areas, road construction in stream beds, deforestation and insufficient drainage structures. Alongside structural measures, watershed management and reduced deforestation were suggested to reduce vulnerability to flood.
[33] (1999)	Multiple Events	U.S.	1968–1995	All	Descriptive analysis of trends in the frequency of extreme events and their associated fatalities and economic losses.	Descriptive analysis (Group B)	Deaths	N.A. (see Section 2.2 and Section 3.5)	No	No	The upward trends in human fatalities and economic losses from extreme events was found to be essentially related to an increased vulnerability stemming from a growing population in coastal areas and lifestyle and demographic (population ageing) changes.
[34] (2012)	Flooding	Vietnam	2007 floods	All	Estimation via contingent valuation of the welfare loss from flood-related illnesses and well-being reduction following flood disaster in a developing country. Willingness to contribute in kind was used to estimate WTP to avoid this welfare loss.	WTP study (Group C)	Flood-related illnesses; well-being reduction	WTC in-kind estimated in the study multiplied by an estimate of the opportunity cost of labour time	No	Yes	Flood damage was estimated on average to represent about 20% of households’ annual income. However, it was not possible to disentangle the welfare loss from morbidity and well-being reduction from the welfare loss due to damages to assets. Poor households were found to be more vulnerable to floods as the associated damage made up a significantly larger portion of their annual income. Households heavily dependent on agricultural activities were also found to be more vulnerable.
[35] (2010)	Extreme temperatures	Taiwan	1971–2006	All	Estimation of (i) the impact of climatic conditions on cardiovascular deaths in Taiwan over 1971–2006 and (ii) WTP (contingent valuation method) to avoid the increase in cardiovascular deaths projected under climate change.	WTP study (Group C)	Cardiovascular deaths	WTP estimated in the study.	No	No	Cardiovascular deaths are projected to increase by 1.2% to 4.1% in Taiwan under alternative IPCC climate scenarios, and each individual would be willing to pay annually $51 to $97 to avoid such an increase in mortality risk.
[36] (2015)	Flooding	Thailand	2011 floods	All	Survey of flood victims in three-severely affected provinces of Thailand to capture health-related and non-health related costs of damage.	Population-based survey (Group D)	Flood-related diseases	Healthcare costs from flood-related diseases	No	Yes	Health-related costs were negligible in contrast to losses to tangible assets (property, valuable etc). Few households experienced health-related losses (11% of sample). Evacuation rates varied between poor and non-poor households: 65% of poor households had some members evacuate vs. 77% for non-poor households.
[37] (2014)	Flooding	Pakistan	Pakistan floods in 2010	All	Comparison of the economic impacts and time-to-recovery after floods in Pakistan versus after an earthquake in Haiti using cross-sectional cluster surveys.	Population-based survey (Group D)	Death and injuries	N.A. (see Section 2.2 and Section 3.5)	No	No	Injuries and deaths were much greater in Haiti. Whilst a decline in income was widespread in both countries, relative household income loss was greater in Pakistan because of damages to the agricultural economy. Housing recovery was however quicker in Pakistan, and food insecurity was smaller than in Haiti, due to greater receipt of food aid.
[38] (2004)	Extreme temp.	U.S.	1995–1998	≥65 years old	Retrospective statistical analysis of the effectiveness of Philadelphia’s heat warning system (PWWS) in terms of reduced excess mortality.	Economic appraisal (Group E)	Deaths	VSL ($4 m)	No	No	117 lives are expected to have been “saved” (with substantial uncertainty around this estimate) over the 3-year period thanks to PWWS. This is equivalent to a gross benefit of $468 m that is much higher than the cost of running the system ($210 k).
[39] (2013)	Hurricanes & tornadoes	U.S.	1989–2005	All	Testing for a potential relationship between hurricane and tornadoes-related casualties and work routine.	“Other” (Group F)	Deaths and injuries	N.A. (see Section 2.2 and Section 3.5)	No	Yes	Daily variation in casualties from hurricanes and tornadoes is affected by the work routine. All things being equal, hurricanes, which provide the at-risk population with some lead time, lead to greater casualties during weekdays since the opportunity cost (namely income loss) of adopting protection measures (e.g., evacuating) is much larger than during weekends. On the opposite, tornadoes, which provide little lead-time, lead to larger casualties during weekends as the acquisition of risk information is harder on week-ends and workplaces and schools are safer than private homes. Casualty risk from tornadoes was found to reduce by 6%–8% for every $1000/per capita income added at the county level.

**Table 2:** Distribution of studies according to their primary environmental hazard and geographical region of focus.

Region	Extreme Temperatures	Floods	Hurricanes	Pollution Peaks	Multiple Events ${}^{3}$	Total by Region
Asia	1	4		2		7
Australia	1					1
Europe	1					1
Turkey ${}^{1}$		2				2
North America ${}^{2}$	4		3		2	9
Total	7	6	3	2	2	20

^1^ As Turkey is both in Europe and Asia it was considered a region of its own; ^2^ all studies in North America were from the U.S.; ^3^ when two or more different types of extreme events were considered in a study.
